# A trial of structured debate as a self‐learning method for students and young healthcare providers to discuss social issues in general and family medicine: A case report in Japan

**DOI:** 10.1002/jgf2.706

**Published:** 2024-05-27

**Authors:** Tatsuki Ikejiri, Jeonse Lee, Natsuki Yokoyama, Arisa Hakariya, Yuki Otsuka, Hayase Hakariya

**Affiliations:** ^1^ Laboratory for Human Nature Cultures and Medicine Kyoto Japan; ^2^ School of Medicine Kyoto Prefectural University of Medicine Kyoto Japan; ^3^ Department of Pharmacy Chubu Tokushukai Hospital Okinawa Japan; ^4^ Minami Seikyo Hospital Nagoya‐shi Aichi Japan; ^5^ Department of General Medicine Okayama University Graduate School of Medicine, Dentistry and Pharmaceutical Sciences Okayama Japan; ^6^ Interfaculty Institute of Biochemistry University of Tuebingen Tuebingen Germany

## Abstract

The structured debate as a means of medical education for medical students and young healthcare providers was performed, with diverse topics related to general and family medicine.

To the Editor,

Structured debate is broadly played by university students and graduates as a competition. The educational benefits of debates, such as fostering critical thinking, are acknowledged across various disciplines.^1^ Hence, we proposed using debates as a self‐learning method for medical students and young healthcare providers. Herein, we report a trial where debate serves as a tool for self‐learning in Japan.

Our debate adhered to the systematic procedures outlined by the Japan Debate Association.^2^ Time allocation was slightly modified (Table S1). We held debates using Zoom, with approximately 5–10 voluntary participants: medical students and young healthcare providers. A “plan (agenda)” was collaboratively based on participants' daily observations and interests (Table 1). The participants were divided into two sides: the “pro” (Affirmative side) and the “con” (Negative side), enabling the debate. After the debate, a panel of three judges decided the persuasiveness of each side. The roles of “pro” and “con” were blindly assigned by a third party, irrespective of individuals' opinions on the topic. We performed four debates, as shown below (Table 1). Participants' motivation for these topics is also described.

**TABLE 1:** Details of structured debates conducted by participants.

Debate No.	Date	Agenda	Motivation
1	November 23, 2020	Community pharmacists should be allowed to administer vaccinations	One of the members is a hospital pharmacist. She would like pharmacists to enhance their medical practice skills
2	January 17, 2021	Hospitals should employ “Clown Doctors,” who stay at hospitals to interact with patients through humor and playful activities	A medical student would like hospitals to be more humorous and appealing places
3	February 28, 2021	Medical professionals, in this case doctors, should provide health counseling in the community outside of medical facilities	A doctor wanted the role of medical professions to be more community‐based
4	May 16, 2021	If a terminally ill elderly person living alone at home visits their primary care physician with a complaint of insomnia, they should not be prescribed benzodiazepine (BZD) medications	This is a frequently encountered situation in medical facilities
5	n.a.	Other topics proposed but not implemented Supervised injection sites (SIS) should be introduced in Japan for those who abuse drugs Online consultation should be encouraged from initial consultation Traditional East Asia medication approach should be recommended as complementary therapy for nausea and vomiting associated with chemotherapy in cancer patients	n.a.

After each debate, time for reflection was provided. Summarizing the participants' feedback, we found three distinctive characteristics of learning through structured debate as follows. First, students and early‐career healthcare providers demonstrated their capability of thinking and researching actively (more easily with a single axis) when given the role of agreeing or disagreeing with a topic in which their interest was vague rather than with no axis. Second, developing discussions within a certain structure will foster the ability to think logically. Lastly, the students will be able to have a multifaceted viewpoint through arguments from both sides, rather than from one point of view.

Of note, our structured debate was conducted online. Given the increased attention toward online educational systems in light of the COVID‐19 pandemic, this trial could potentially serve as a promising alternative tool in the future.

As a limitation, our debates were performed without the involvement of leadership experts, as we intended to provide a voluntarily investigating platform to explore topics of participants' interest. Consequently, the quality of the debates depended markedly on the participants' motivation. Moreover, given that participants of this trial were sufficiently motivated at least enough to invest their precious time voluntarily, well‐organized and appealing instructions should be required to install our method in the usual classroom education. For instance, supervision by senior group mentors, who have already acquired the credit, during participants' preparation stage may be beneficial. Prior discussion and consultation with such mentors could also serve as students' gatekeeping not to miss essential points and maintain their direction. Imposing the report submission could also function as a quality assurance opportunity; nonetheless, burdens for teachers would be high.

Additionally, our assessment of the educational effect of the debate is insufficient, given that we solely gathered narrative feedback. However, it is noteworthy that only a few studies evaluated the educational effect of structured debate in the medical field; these reports included a variety of controversial topics such as medical ethics, healthcare systems, and emergency medicine.^3^, ^4^, ^5^ A more comprehensive evaluation from various viewpoints would reinforce our proposal to utilize structured debates as a medical educational platform for students and early‐career medical professionals.

## CONFLICT OF INTEREST STATEMENT

The authors have stated explicitly that there are no conflicts of interest in connection with this article.

## Supporting information


Table S1.
