# Supplementary material for: A trial of structured debate as a self‐learning method for students and young healthcare providers to discuss social issues in general and family medicine: A case report in Japan
Source: J Gen Fam Med. 2024 May 27;25(5):293–4. doi: 10.1002/jgf2.706 (PMC11577296; doi:10.1002/jgf2.706)
Supplement: Supplementary file 1 — Table S1. [file JGF2-25-293-s001.docx]

# **Table S1. Time allocation modified from the Japan Debate Association (JDA)’s rule**

|  | Content | Time |
| --- | --- | --- |
| **AFF*** | Basic Argument | 5 min |
| **NEG*** | Question | 1 min |
| **NEG** | Basic Argument | 5 min |
| **AFF** | Question | 1 min |
| **AFF** | Developmental Argument and Basic Refutation | 4 min |
| **NEG** | Question | 1 min |
| **NEG** | Developmental Argument and Basic Refutation | 4 min |
| **AFF** | Question | 1 min |
| **NEG** | Developmental Refutation | 5 min |
| **AFF** | Developmental Refutation | 5 min |
| **NEG** | Conclusion | 4 min |
| **AFF** | Conclusion | 4 min |
|  |  | Total 40 min |

*AFF; Affirmative side. NEG; Negative side.
